# Unleashing nature's defense: potent antimicrobial power of plant extracts against oral pathogens and Streptococcus mutans biofilms

**DOI:** 10.3389/froh.2024.1469174

**Published:** 2024-12-12

**Authors:** Joachim Hickl, Aikaterini Argyropoulou, Ali Al-Ahmad, Elmar Hellwig, Alexios Leandros Skaltsounis, Annette Wittmer, Kirstin Vach, Lamprini Karygianni

**Affiliations:** ^1^ Department of Operative Dentistry and Periodontology, Medical Center, Faculty of Medicine, University of Freiburg, Freiburg, Germany; ^2^ Department of Pharmacognosy and Natural Products Chemistry, Faculty of Pharmacy, National and Kapodistrian University of Athens, Athens, Greece; ^3^ Institute of Medical Microbiology and Hygiene, Faculty of Medicine, University of Freiburg, Freiburg, Germany; ^4^ Institute for Medical Biometry and Statistics, Faculty of Medicine and Medical Center, University of Freiburg, Freiburg, Germany; ^5^ Clinic of Conservative and Preventive Dentistry, Center of Dental Medicine University of Zurich, Zurich, Switzerland

**Keywords:** Mediterranean herb, natural extract, oral disease, antimicrobial activity, biofilm

## Abstract

**Objectives:**

The increasing demand for alternatives to antibiotics against resistant bacteria has led to research on natural products. The aim of this study was to analyze the antimicrobial and antibiofilm activity of 16 Mediterranean herb extracts.

**Materials and methods:**

The extracts were analyzed using High Performance Thin Layer Chromatography. The minimum inhibitory concentration and minimum bactericidal concentration of the extracts from *Achillea taygetea*, *Cistus creticus* ssp. *creticus*, *Cistus monspeliensis*, *Lavandula stoechas*, *Mentha aquatica*, *Mentha longifolia*, *Origanum vulgare*, *Phlomis cretica*, *Rosmarinus officinalis*, *Salvia sclarea*, *Satureja parnassica*, *Satureja thymbra*, *Sideritis euboea*, *Sideritis syriaca*, *Stachys spinosa*, and *Thymus longicaulis* were determined against eight oral bacteria and fungus *Candida albicans*. Microtiter plate test was conducted to evaluate the antibiofilm activity against *Streptococcus mutans*.

**Results:**

Overall, all tested extracts efficiently suppressed the growth of obligate anaerobic bacteria. When applied at concentrations ≥0.15 mg/ml, the extracts exhibited moderate to high antibiofilm activity comparable to that of chlorhexidine (CHX) against *S. mutans*. Interestingly, *R. officinalis* (MIC: 0.01–0.06 mg/ml) and *O. vulgare* (MIC: 0.04–1.25 mg/ml) demonstrated the highest antibacterial activity against oral bacteria. Additionally, *R. officinalis* and *L. stoechas* significantly inhibited *S. mutans* biofilm formation at 0.15 mg/ml.

**Conclusions:**

The tested plant extracts can be considered as alternative natural antimicrobial and antibiofilm agents.

**Clinical relevance:**

Mediterranean herb extracts show promise as natural alternatives to combat oral bacteria and biofilm formation, offering potential new therapies for infectious oral diseases in the context of antibiotic resistance.

## Introduction

Over the past two decades, the rise in antimicrobial resistance among pathogenic bacteria has significantly contributed to the persistence of various bacterial infections in the human body. The current research on antimicrobials in the medical field has confirmed the widespread occurrence of antimicrobial resistance, leading to a crisis in antimicrobial resistance (1, 2). In dentistry, the commonly used local disinfectant chlorhexidine (CHX) not only exhibits toxic effects on host cells but also possesses the ability to promote antimicrobial resistance/tolerance through mechanisms such as bacterial membrane alteration, resistance genes, and multidrug efflux pumps (3, 4). Chlorhexidine (CHX) is highly effective against biofilms formed by *Streptococcus mutans* and other oral bacteria, but its limitations, including the potential for increased bacterial tolerance, highlight the need for alternative antibiofilm agents such as plant extracts (5, 6). Another challenge in combating oral biofilms, which are up to 1,000 times less susceptible to conventional antimicrobial agents compared to their planktonic counterparts, is the eradication of bacteria residing in the deep layers of these biofilms (7, 8).

In the oral cavity, there are approximately 700 known bacterial species that colonize various surfaces, including the gingiva, teeth, and other oral mucosal sites (9). These bacteria form highly organized microbial communities called biofilms, which provide them with significant protection against antimicrobial agents. The biofilm structure hinders the diffusion of antimicrobial agents (10), and the deepest layers of the biofilm have reduced oxygen levels and a low rate of cell division (11). During the formation of oral biofilms, the initial attachment occurs on the pellicle, a layer primarily composed of salivary proteins. Early colonizers, such as *Streptococcus* spp., *Actinomyces* spp., *Veillonella* spp., and *Neisseria* spp., adhere to the pellicle (12, 13). Subsequently, *Fusobacterium nucleatum* creates a microenvironment with reduced oxygen levels, favoring the adhesion of strict anaerobic pathogens, including late colonizers such as *Aggregatibacter actinomycetemcomitans*, *Porphyromonas gingivalis*, and *Prevotella intermedia* (14).

Mediterranean plants have been extensively studied as valuable natural resources for medicinal purposes (15). Over time, numerous biochemical compounds derived from these plants have been identified (16). Further investigations into developing plant-derived antibiotics have highlighted the antimicrobial properties of various compounds, including phenolic acids, flavonoids, plant peptides, phenanthrenes, and terpenes (17–20). Plant metabolites such as phenolics, terpenoids, sulfur-containing compounds, coumarins, quinones, and alkaloids have shown significant biological activity as anti-biofilm agents and inhibitors of quorum sensing (21). Plant-derived extracts offer several advantages over traditional antimicrobials (22). These extracts are less likely to induce bacterial resistance because they contain a complex mixture of bioactive compounds and can target multiple pathways in bacteria. They also tend to be less toxic to human tissues (23, 24). Furthermore, many plant compounds have anti-inflammatory and antioxidant properties, which can benefit oral health by not only inhibiting bacterial growth, but also promoting tissue healing and reducing inflammation associated with infections (25).

Regional plant products are utilized worldwide, particularly in impoverished nations where they serve as the most affordable form of medicine. However, it is crucial to optimize their formulation and development by targeting specific molecular mechanisms (26). These plant products have the potential to enhance oral health (27) and overall systemic well-being. Several plant-based products have been utilized in oral healthcare and medicinal formulations to combat dental caries, periodontitis, and gingivitis (28). The treatment of pathogenic microbes, which pose significant challenges due to their pathogenicity and resistance, can be improved by targeting them when they are more susceptible to alternative natural antibiotics (29). Considering the complexity of polymicrobial interactions and the intricate compositions of plant-derived products, it is crucial to conduct further research on additional plant species, extraction methods, and explore the synergistic effects of different compounds. Urgent investigation in these areas is necessary.

The objective of this report was to investigate the antimicrobial activity of various Mediterranean herb extracts against different microorganisms. Specifically, the ethyl acetate extracts of *Achillea taygetea*, *Cistus creticus*, *Cistus monspeliensis*, *Lavandula stoechas*, *Mentha aquatica*, *Mentha longifolia*, *Origanum vulgare*, *Phlomis cretica*, *Rosmarinus officinalis*, *Salvia sclarea*, *Satureja parnassica*, *Satureja thymbra*, *Sideritis euboea*, *Sideritis syriaca*, *Stachys spinosa*, and *Thymus longicaulis* were tested against eight common oral pathogenic bacteria and the fungus *Candida albicans*. Additionally, two reference strains, *Staphylococcus aureus* and *Escherichia coli*, which are found on the skin and intestinal mucosa, respectively, were included in the study. Some extracts were previously evaluated for antimicrobial activity but not on oral pathogens specifically, highlighting the novelty of our findings on their inhibitory effects on biofilm formation.

The null hypothesis states that the aforementioned extracts do not exhibit any antimicrobial effects on the tested microbial species. To test this hypothesis, three antimicrobial assays were conducted: the minimal inhibitory concentration (MIC) assay, the minimal bactericidal concentration (MBC) assay, and the biofilm plate assay.

## Materials and methods

### Extraction process

Aerial components of sixteen distinct plant species were gathered from diverse locations within the Greek periphery. The plant species encompassed *Achillea taygetea* Boiss. & Heldr., *Cistus creticus* L., *Cistus monspeliensis* L., *Lavandula stoechas* L., *Mentha aquatica* L., *Mentha longifolia* L., *Origanum vulgare* L., *Phlomis cretica* C. Presl, *Rosmarinus officinalis* L., *Salvia sclarea* L., *Satureja parnassica* Heldr. & Sart. ex Boiss., *Satureja thymbra* L., *Sideritis euboea* Heldr., *Sideritis syriaca* L., *Stachys spinosa* L., and *Thymus longicaulis* C. Presl. The collected plant specimens were finely ground (using SCIS, Allenwest-Eac ltd) into homogeneous powders and subjected to ultrasound-assisted extraction (UAE). An Elma S 100H (Elmasonic) instrument was employed, utilizing 100% ethyl acetate as the extraction solvent. The extraction process took place for 15 min at room temperature, with a plant-to-solvent ratio of 1/10 (w/v). To ensure comprehensive extraction, the procedure was repeated twice for each sample. The ethyl acetate solvent was subsequently evaporated to dryness under reduced pressure, employing a Buchi Rotavapor R-200 rotary evaporator, while maintaining a temperature of 40°C.

### High performance thin layer chromatography (HPTLC) analysis

For the creation of fingerprint profiles of the diverse extracts, an instrumental setup of Camag HPTLC was employed. Solutions of the extracts were prepared by dissolving 10 mg of each extract in 1 ml of ethyl acetate. To apply plant extract samples onto TLC plates measuring 20 × 10 cm (silica gel 60, F254, Merck), the Automatic TLC Sampler (ATS4, CAMAG) was utilized. This process was controlled through the VisionCats 2.3 software platform (Camag), following standardized configurations: 6 tracks featuring 8 mm bands, an 8 mm distance from the lower edge, 20 mm from both the left and right edges, and a 10.4 mm spacing between distinct tracks. Each sample was applied with an 8 μl volume. The ensuing plate development was conducted in an automatic development chamber (ADC2), adhering to established norms: a 20-minute chamber saturation period using filter paper, followed by 10 min of plate conditioning at 33% relative humidity (using MgCl2), and concluding with a 5-minute plate drying phase. Toluene/ethyl acetate/formic acid (80:20:2; v/v/v) were chosen as the mobile phases. Imaging at both 254 nm and 366 nm was captured using a Visualizer 2 Documentation System (CAMAG, Muttenz, Switzerland).

### Bacterial and fungal strains

Ten bacterial strains and one *Candida albicans* strain were specifically chosen for this study. Among these, eight bacterial strains and the *Candida albicans* strain are considered typical inhabitants of the oral cavity. In contrast, *Staphylococcus aureus* is primarily associated with the skin, while *Escherichia coli* is commonly found within the intestinal flora. These two species, *S. aureus* and *E. coli*, were incorporated as reference strains for comparison. Within the selected strains, *Streptococcus mutans* DSM 20523, *Streptococcus sobrinus* DSM 20381, *Streptococcus oralis* ATCC 35037, *Enterococcus faecalis* ATCC 29212, and *S. aureus* ATCC 25923 represent facultative anaerobic Gram-positive species. Notably, *E. coli* ATCC 25922, possessing a Gram-negative cell wall, is also facultative anaerobic. On the other hand, *Porphyromonas gingivalis* W381, *Prevotella intermedia* MSP34 (a clinical isolate), *Fusobacterium nucleatum* ATCC 25586, and *Parvimonas micra* ATCC 23195 are categorized as obligate anaerobic bacteria. The sole fungal species employed, *C. albicans* DSM 1386, is capable of growth in both yeast and filamentous forms. All the bacterial and fungal strains were graciously provided by the Division of Infectious Diseases and the Institute of Medical Microbiology and Hygiene at Albert-Ludwigs-University in Freiburg. These microorganisms were stored at −80°C in a basic growth medium supplemented with 15% (v/v) glycerol until their utilization in the study.

### Determination of the minimum inhibitory concentration (MIC)

Initially, an overnight culture for every bacterial and fungal strain was prepared following the Clinical and Laboratory Standards Institute (CLSI) guidelines. Each microorganism was plated onto Columbia blood agar plates (CBA) or yeast-cysteine blood agar plates (HCB). Facultative anaerobic bacteria and *Candida albicans* were cultivated on CBA agar plates at 37°C in a 5%–10% CO_2_ atmosphere for 24 h. Meanwhile, the anaerobic bacteria were plated on HCB agar plates and incubated at 37°C for 48 h within an anaerobic chamber (Anaerocult, Merck Chemicals GmbH, Darmstadt, Germany). A 0.5 A/1 A McFarland standard suspension was generated in 0.9% saline (NaCl) for facultative anaerobic bacteria and *Candida albicans*, respectively. For the microdilution assay, all facultative anaerobic strains and *Candida albicans* were subsequently 1:10 diluted in BBL™ Mueller Hinton II Broth-Cation-Adjusted (MHB, BD, Heidelberg, Germany). The anaerobic bacteria were prepared in Wilkins–Chalgren broth (WCB) at a 0.5 A McFarland standard suspension. As stipulated by ISO 20776-1:2006, tests involving facultative anaerobic bacteria required a cell density of approximately 5 × 10^^5^ colony forming units (CFU) per ml, while fungi tests utilized 5 × 10^^4^ CFU/ml, and tests involving obligate anaerobic bacteria used 5 × 10^^6^ CFU/ml. Subsequently, suitable volumes of the MHB/WCB microbial cultures were transferred into a 96-well microtiter plate using a multi-channel pipette. The prepared natural plant extracts were dissolved in dimethyl sulfoxide (DMSO, Sigma-Aldrich Chemie GmbH, Steinheim, Germany) at a concentration of 100 mg/ml. Concentration series of extract solutions in DMSO ranged from 10 mg/ml to 0.02 mg/ml, employing dilution levels from 10-fold to 5120-fold. Each well in the 96-well microtiter plate held a total volume of 100 µl. To rule out any potential antimicrobial effects of residual DMSO, a parallel dilution series of DMSO was investigated. Wells containing solely MHB/WCB, as well as a dilution series of 0.1% chlorhexidine (CHX), served as negative and positive controls for bacterial growth, respectively. Additionally, wells containing MHB/WCB and the added microbial strain were designated as growth controls. Contamination risks were minimized through the use of sterile MHB/WCB. Subsequently, *E. coli*, *S. aureus*, *E. faecalis*, and *C. albicans* were incubated at 37°C for 18 h, while the three streptococci strains were incubated at 37°C under a 5%–10% CO2 atmosphere for 24 h. Anaerobic bacteria were maintained at 37°C for 48 h within an anaerobic jar (Anaerocult, Merck Chemicals GmbH, Darmstadt, Germany). All assays for each bacterial and fungal strain were carried out in duplicates, and the highest minimum inhibitory concentration (MIC) values were considered if MIC values exhibited minor discrepancies. If differences between two rows exceeded two dilution levels, the determination involving that specific extract was repeated. MIC was defined as the lowest concentration of each natural plant extract that visibly inhibited bacterial growth. The inhibitory effect of DMSO was taken into account if bacterial growth was observed within the co-tested DMSO dilution series.

### Determination of the minimum bactericidal concentration (MBC)

The assessment of the minimum bactericidal concentration (MBC) was also conducted following the protocols outlined by the CLSI. Following the completion of the MIC assay, the 96-well microtiter plates were subjected to further incubation for MBC testing. In a concise manner, 10 µl from each well, containing the respective concentration series of the tested plant extracts, were plated onto CBA or HCB plates. Specifically, *E. coli*, *S. aureus*, and *E. faecalis* were plated on CBA agar plates and then incubated at 37°C for 24 h. Streptococci and *C. albicans* were placed on CBA agar plates and incubated at 37°C in a 5%–10% CO_2_ atmosphere for 2 days. On the other hand, the obligate anaerobes were cultivated on HCB agar plates at 37°C for a duration of 5 days within an anaerobic chamber (Anaerocult, Merck Chemicals GmbH, Darmstadt, Germany). Ultimately, a visual determination of colony-forming units (CFU) was performed. The MBC was defined as the concentration at which a three-log decrease in bacterial growth (equivalent to 99.9% inhibition) was observed in comparison to the growth control.

### Biofilm plate assay

Initially, an overnight cultivation of the *S. mutans* R15-8 bacterial strain (a clinical isolate) was performed at 37°C under aerobic conditions with a 5%-10% CO_2_ atmosphere in BMH (BD, Heidelberg, Germany) supplemented with 1% sucrose (MH-S). Following this, polystyrene 96-well tissue-culture plates (Greiner bio-one, Frickenhausen, Germany) were loaded with 100 µl of MH-S, incorporating ten distinct concentrations (ranging from 0.019 mg/ml to 10 mg/ml) of the plant extracts under investigation. Subsequently, 5 µl of the *S. mutans* overnight culture were added to each well. The Log_10_ of the CFU of the *S. mutans* overnight culture on CBA plates fell within the range of 10^8^ CFU/ml. These 96-well plates were then incubated for 48 h at 37°C in an aerobic environment with a 5%-10% CO_2_ atmosphere. Following the incubation period, the culture medium was discarded, and the wells were subjected to three consecutive washes using 300 ml of phosphate-buffered saline (PBS, Life Technologies, Darmstadt, Germany) per plate in order to eliminate non-adherent bacteria. Since no fixation of adherent bacterial cells within the biofilm was deemed necessary, the plates were simply air-dried and subsequently stained with Carbol Gentian Violet solution (Carl Roth GmbH + Co. KG, Karlsruhe, Germany) intended for microscopy. This staining solution contained 0.1%–<0.25% methyl violet and was applied for a duration of 10 min. After staining, excess dye was washed away by rinsing the plates with distilled water. The plates were then dried at 60°C for 10 min. To facilitate dye resolubilization, 50 µl of absolute ethanol (99.9 vol%) was added to each well for subsequent analysis (Merck Chemicals GmbH, Darmstadt, Germany), and the optical density was finally measured at 595 nm using the Tecan Infinite 200 plate reader (Tecan, Crailsheim, Germany). All experimental tests were executed in quadruplicate, and the mean values were subsequently calculated. To validate the findings and further eliminate false positive results, the plant extracts yielding the highest biofilm inhibition values underwent a second screening. During the analysis, the antibiofilm effects of each extract on *S. mutans* were classified into three distinct groups, aided by two different cut-off values: no biofilm production or C1, moderate biofilm production or C2, and high biofilm production or C3. The low cut-off value was established by adding three standard deviations of the blank to the negative control. Conversely, the high cut-off value was derived after conducting the low cut-off value measurement on three separate occasions. The low and high cut-off OD595 values were estimated at 0.143 and 0.428, respectively. High *S. mutans* biofilm inhibition is exhibited at OD595 values ≤ 0.143, whereas *S. mutans* moderate biofilm formation is displayed at 0.143 ≤ OD595 values ≤0.428. DMSO and CHX concentrations are shown for each extract concentration.

### Statistical analysis

For descriptive analysis median values, mean values and standard deviations were computed. *T*-tests were applied between the logarithmic adsorption values (basis 10) of the extracts and the two control groups, respectively, with a Bonferroni-correction due to multiple testing. For graphical presentation of the results scatter plots were used. All computations were done with STATA (Version 17.0, College Station, TX, USA).

## Results

Continuing our attempt to find new plant extracts with potential inhibition activity against oral microorganisms, sixteen extracts from various genus were selected to be screened. The plants belonged to three families Lamiaceae, Cistaceae and Asteraceae and are commonly encountered in the Mediterranean region. However, their antimicrobial activity against typical oral pathogenic bacteria hasn't been studied before. Following the extraction of the plant samples with ethyl acetate a rapid and accurate analytical method was developed, aiming to the detection of the major active compounds in the extracts. HPTLC analysis of the ethyl acetate extracts revealed that the most of the plants have a rich chemical content. Plants belonging to the Lamiaceae family contain a wide range of bioactive compounds and are well known for their antibacterial, antifungal and antioxidant properties. They are among the most actively used in phytotherapy and are considered important for the pharmaceutical, food and cosmetic industries. *Lavandula stoechas*, *Mentha aquatica*, *Mentha longifolia*, *Origanum vulgare*, *Phlomis cretica*, *Rosmarinus officinalis*, *Salvia sclarea*, *Satureja parnassica*, *Satureja thymbra*, *Sideritis euboea*, *Sideritis syriaca*, *Stachys spinosa* and *Thymus longicaulis*are well studied plants for their biological properties. Visualization of the plates at 254 nm and 366 nm revealed the presence of phenolic compounds, like the phenolic acid caffeic acid, and mainly flavonoid aglycones, such as apigenin and luteolin. The presence of rosmarinic acid was evident in *Lavandula*, *Mentha*, *Origanum*, *Rosmarinus*, *Salvia* and *Thymus*. *Achillea* sp. is a genus of the well known medicinal plant family of Asteraceae and comprises numerous species and wild-growing plants. *A. taygetea* is endemic at the mountains Taygetos and Parnon (south Peloponnese). The plant was also extracted and its analysis showed that it contains various flavonoids, derivatives of apigenin and luteolin, and phenolic acids. *Cistus creticus* and *Cistus monspeliensis* are medicinal plants that belong to the Cistaceae family, with a well-established position in traditional medicine of the Mediterranean basin. Similarly, several secondary metabolites flavonoids and phenolic acids were identified in the extracts as major components (see Supplementary Figure S1).

### Achillea taygetea

The ethyl acetate extract of *A. taygetea* demonstrated significant inhibitory effects on obligate anaerobic pathogens, with MIC values ranging from 0.04 mg/ml (*P. micra*) to 0.60 mg/ml (*F. nucleatum*). The effect on facultative anaerobic streptococci varied, with *S. oralis* inhibited at 0.60 mg/ml, *S. sobrinus* inhibited at a minimum of 5.00 mg/ml, and the inhibitory concentrations observed in *S. mutans* tests were attributed to the effects of DMSO. Among the remaining pathogens listed in Table 1, all except *S. aureus* (MIC = 2.50 mg/ml) showed resistance to the extract. The MBC values ranged from 0.15 mg/ml (*P. micra*) to 5.00 mg/ml (*S. aureus*), except for *S. sobrinus*, which was not eliminated in the test.

**Table 1:** Antimicrobial activity in mg ml^−1^ of *Achillea taygetea* ethyl acetate extract.

Achillea taygetea				
Sample	Ethyl acetate extract DMSO (%)			
c/mg ml**^−^**^1^	MIC	MBC	MIC	MBC
*Streptococcus* mutans DSM 20523	2.50	NA	5.00	NA
*Streptococcus sobrinus* DSM 20381	5.00	NA	20.00	NA
*Streptococcus oralis* ATCC 35037	0.60	0.60	10.00	20.00
*Enterococcus faecalis* ATCC 29212	NA	NA	20.00	NA
*Candida albicans* DSM 1386	10.00	10.00	10.00	NA
*Escherichia coli* ATCC 25922	10.00	NA	20.00	NA
*Staphylococcus aureus* ATCC 25923	2.50	5.00	20.00	NA
*Porphyromonas gingivalis* W381	0.15	0.30	20.00	20.00
*Prevotella intermedia* MSP 34	0.15	0.30	5.00	5.00
*Fusobacterium nucleatum* ATCC 25586	0.60	0.60	10.00	10.00
*Parvimonas micra* ATCC 23195	0.04	0.15	5.00	20.00

NA, No activity observed: MIC or MBC of extracts were measured at 10.00 mg ml^−1^ and DMSO at 20%, respectively.

MIC = extract concentration at which the optical density (OD) measurement revealed minimal bacterial growth.

MBC = extract concentration at which a three-log reduction (99.9%) of the bacterial growth was induced.

In the biofilm plate assay, the tested *S. mutans* strain showed strong inhibition in biofilm formation at a concentration of 5.00 mg/ml. The lower cutoff value was calculated at an optical density (OD595) of 0.143. In the presence of 2.50 mg/ml of the ethyl acetate extract, biofilm production fell into the C2 category. Figure 2 indicates that lower concentrations did not affect biofilm formation, as all absorbance values were higher than the high cutoff value (OD_595_ = 0.428).

### Cistus criticus and Cistus monspeliensis

The mean MIC and MBC values for the ethyl acetate extract against the tested bacterial and fungal strains are summarized in Table 2. The MIC values for the inhibited bacterial strains ranged from 0.04 mg/ml (*P. gingivalis*, *P. micra*) to 5.00 mg/ml (*S. sobrinus*). The MBC values indicated that 99.9% of the bacterial strains were killed at concentrations ranging from 0.04 mg/ml (*P. micra*) to 5.00 mg/ml (*S. aureus*). *E. faecalis* (MIC = 2.50 mg/ml) and *S. sobrinus* were inhibited but not completely eradicated in the test. *S. mutans*, *E. coli*, and *C. albicans* did not appear to be significantly affected by the extract.

**Table 2:** Antimicrobial activity in mg ml^−1^ of *Cistus creticus* ethyl acetate extract.

Cistus creticus				
Sample	Ethyl acetate extract DMSO (%)			
c/mg ml**^−^**^1^	MIC	MBC	MIC	MBC
*Streptococcus mutans* DSM 20523	5.00	NA	10.00	NA
*Streptococcus sobrinus* DSM 20381	5.00	NA	20.00	NA
*Streptococcus oralis* ATCC 35037	0.15	0.60	20.00	20.00
*Enterococcus faecalis* ATCC 29212	2.50	NA	20.00	NA
*Candida albicans* DSM 1386	10.00	10.00	10.00	NA
*Escherichia coli* ATCC 25922	NA	NA	20.00	NA
*Staphylococcus aureus* ATCC 25923	1.25	5.00	20.00	NA
*Porphyromonas gingivalis* W381	0.04	0.08	20.00	20.00
*Prevotella intermedia* MSP 34	0.15	0.30	5.00	5.00
*Fusobacterium nucleatum* ATCC 25586	0.60	0.60	10.00	10.00
*Parvimonas micra* ATCC 23195	0.04	0.04	5.00	20.00

NA, No activity observed: MIC or MBC of extracts were measured at 10.00 mg ml^−1^ and DMSO at 20%, respectively.

MIC = extract concentration at which the optical density (OD) measurement revealed minimal bacterial growth.

MBC = extract concentration at which a three-log reduction (99.9%) of the bacterial growth was induced.

Similar to the MIC/MBC assays, the biofilm plate assay showed no detectable effect on biofilm formation (C3) by *S. mutans*, as depicted in Figure 3.

The ethyl acetate extract exhibited potent antimicrobial activity against oral pathogens, with MBC values ranging from 0.04 mg/ml (*P. micra*) to 5.00 mg/ml for *E. faecalis*. It also demonstrated significant activity against *C. albicans*. However, except for the antimicrobial effect of DMSO (as shown in Table 3), the extract had no impact on *E. coli*. The most pronounced inhibitory effect was observed at 0.04 mg/ml on *P. micra*, closely followed by 0.08 mg/ml for *S. oralis* and *P. gingivalis*. The mean minimum inhibitory concentration (MIC) and MBC values for the typical skin microbe *S. aureus* were 0.60 mg/ml.

**Table 3:** Antimicrobial activity in mg ml^−1^ of *Cistus monspeliensis* ethyl acetate extract.

Cistus monspeliensis				
Sample	Ethyl acetate extract DMSO (%)			
c/mg ml**^−^**^1^	MIC	MBC	MIC	MBC
*Streptococcus mutans* DSM 20523	2.50	2.50	5.00	NA
*Streptococcus sobrinus* DSM 20381	2.50	2.50	10.00	NA
*Streptococcus oralis* ATCC 35037	0.08	0.08	20.00	20.00
*Enterococcus faecalis* ATCC 29212	5.00	5.00	20.00	NA
*Candida albicans* DSM 1386	5.00	5.00	10.00	20.00
*Escherichia coli* ATCC 25922	NA	NA	20.00	NA
*Staphylococcus aureus* ATCC 25923	0.60	0.60	20.00	NA
*Porphyromonas gingivalis* W381	0.08	0.08	20.00	20.00
*Prevotella intermedia* MSP 34	0.30	0.30	2.50	2.50
*Fusobacterium nucleatum* ATCC 25586	0.30	0.30	5.00	10.00
*Parvimonas micra* ATCC 23195	0.04	0.04	5.00	10.00

NA, No activity observed: MIC or MBC of extracts were measured at 10.00 mg ml^−1^ and DMSO at 20%, respectively.

MIC = extract concentration at which the optical density (OD) measurement revealed minimal bacterial growth.

MBC = extract concentration at which a three-log reduction (99.9%) of the bacterial growth was induced.

Figure 3 indicates that the biofilm formation of *S. mutans* was not influenced by the tested concentrations of the ethyl acetate extract.

#### Lavandula stoechas

Table 4 provides an overview of the MIC and MBC values for the ethyl acetate extract of *Lavandula stoechas*. Overall, the extract exhibited significant inhibitory effects on obligate anaerobic bacteria, with MIC values ranging from 0.04 mg/ml (*P. gingivalis*) to 1.25 mg/ml (*F. nucleatum*). Similarly, it displayed strong inhibitory effects on facultative anaerobic oral bacteria, with MIC values ranging from 0.15 mg/ml (*S. oralis*, *S. mutans*) to 1.25 mg/ml (S*. sobrinus*). The extract did not demonstrate antibacterial effects against *E. coli* and antifungal effects against *C. albicans*, except for the effects of DMSO.

**Table 4:** Antimicrobial activity in mg ml^−1^ of *Lavandula stoechas* ethyl acetate extract.

Lavandula stoechas				
Sample	Ethyl acetate extract DMSO (%)			
c/mg ml**^−^**^1^	MIC	MBC	MIC	MBC
*Streptococcus mutans* DSM 20523	0.15	NA	5.00	NA
*Streptococcus sobrinus* DSM 20381	1.25	NA	20.00	NA
*Streptococcus oralis* ATCC 35037	0.15	0.30	10.00	20.00
*Enterococcus faecalis* ATCC 29212	0.60	1.25	20.00	NA
*Candida albicans* DSM 1386	10.00	NA	10.00	NA
*Escherichia coli* ATCC 25922	NA	NA	20.00	20.00
*Staphylococcus aureus* ATCC 25923	1.25	1.25	20.00	NA
*Porphyromonas gingivalis* W381	0.04	0.15	20.00	20.00
*Prevotella intermedia* MSP 34	0.30	1.25	5.00	5.00
*Fusobacterium nucleatum* ATCC 25586	1.25	2.50	5.00	10.00
*Parvimonas micra* ATCC 23195	0.08	0.60	2.50	20.00

NA, No activity observed: MIC or MBC of extracts were measured at 10.00 mg ml^−1^ and DMSO at 20%, respectively.

MIC = extract concentration at which the optical density (OD) measurement revealed minimal bacterial growth.

MBC = extract concentration at which a three-log reduction (99.9%) of the bacterial growth was induced.

While most oral bacteria showed at least a low bactericidal effect, with MBC values ranging from 0.15 mg/ml (*P. gingivalis*) to 2.50 mg/ml (*F. nucleatum*), a measurable MBC for *S. mutans* and *S. sobrinus* was not observed. *S. aureus* was inhibited by the extract, and 99.9% of the bacteria were killed at a concentration of 1.25 mg/ml.

**Figure 1:**
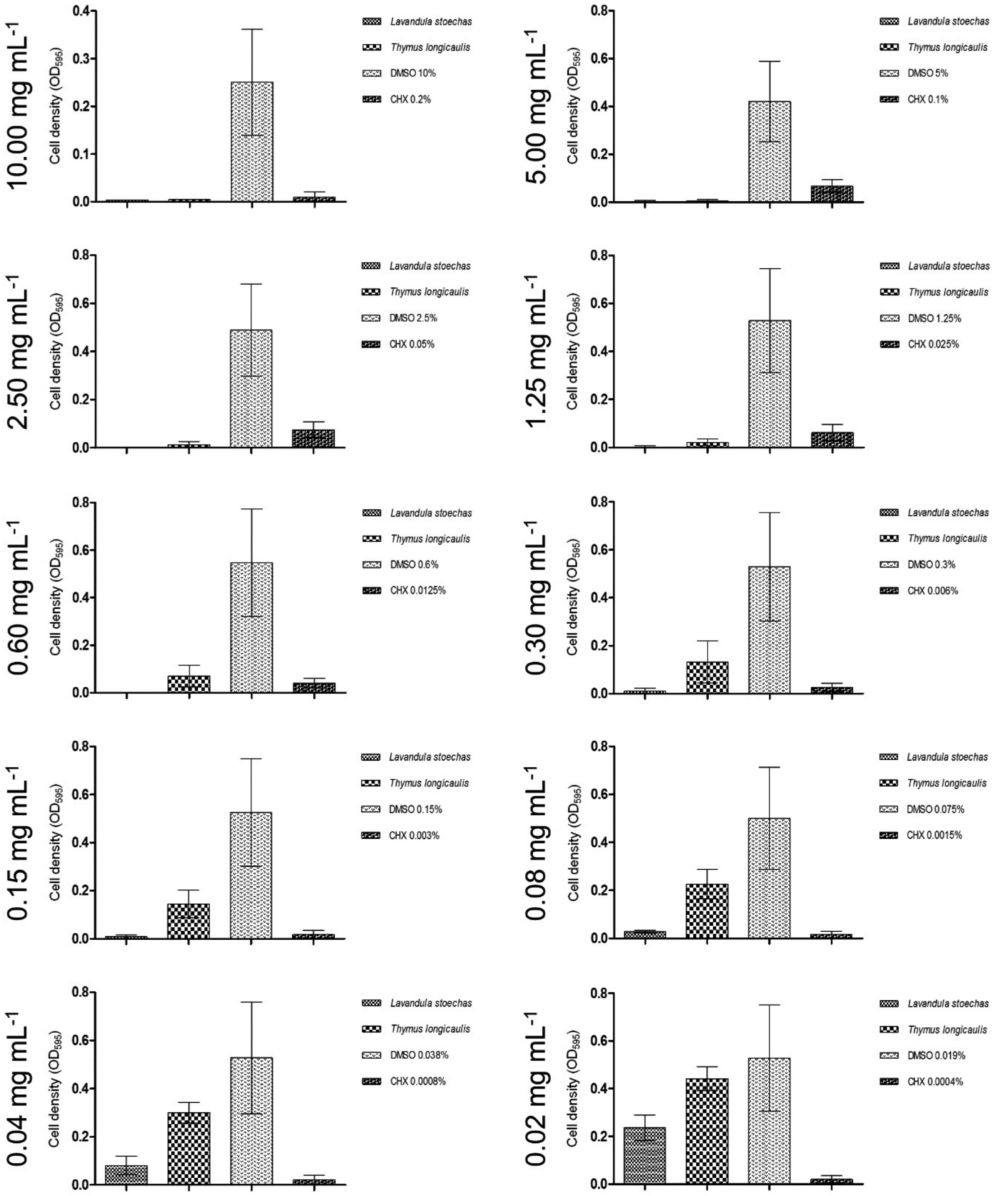
Graphs demonstrating ethyl acetate extracts with high impact on biofilm inhibition.

According to Figure 1, the ethyl acetate extract of *L. stoechas* exhibited significant inhibition of biofilm formation, even at concentrations as low as 0.04 mg/ml. The lowest tested concentration of 0.02 mg/ml can still be categorized as having a moderate inhibitory effect (C2) on biofilm formation.

#### Mentha aquatica and Mentha longifolia

Table 5 presents the MIC and MBC values for the ethyl acetate extract of *Mentha aquatica*. The extract demonstrated efficient reduction of obligate anaerobic bacteria, with inhibitory concentrations ranging from 0.30 mg/ml (*P. gingivalis*) to 1.25 mg/ml (*F. nucleatum*). With the exception of *S. oralis* and *C. albicans*, all other pathogens exhibited slight inhibition, with MIC values ranging from 2.50 mg/ml (*S. mutans*, *E. faecalis*) to 5.00 mg/ml (*S. sobrinus*, *E. coli*, *S. aureus*). Furthermore, the extract displayed a moderate bactericidal effect on *P. micra*, *P. gingivalis*, and *S. oralis*, with MBC values ranging from 1.25 mg/ml (*P. micra*) to 5.00 mg/ml (*S. oralis*). When considering the effects of DMSO, no significant bactericidal effect was observed on the other strains.

**Table 5:** Antimicrobial activity in mg ml^−1^ of *Mentha aquatica* ethyl acetate extract.

Mentha aquatica				
Sample	Ethyl acetate extract DMSO (%)			
c/mg ml**^−^**^1^	MIC	MBC	MIC	MBC
*Streptococcus mutans* DSM 20523	2.50	NA	10.00	NA
*Streptococcus sobrinus* DSM 20381	5.00	NA	20.00	NA
*Streptococcus oralis* ATCC 35037	5.00	5.00	10.00	20.00
*Enterococcus faecalis* ATCC 29212	2.50	NA	20.00	NA
*Candida albicans* DSM 1386	5.00	NA	10.00	20
*Escherichia coli* ATCC 25922	5.00	NA	20.00	NA
*Staphylococcus aureus* ATCC 25923	5.00	NA	20.00	NA
*Porphyromonas gingivalis* W381	0.30	2.50	20.00	20.00
*Prevotella intermedia* MSP 34	0.60	2.50	5.00	5.00
*Fusobacterium nucleatum* ATCC 25586	1.25	5.00	10.00	10.00
*Parvimonas micra* ATCC 23195	0.60	1.25	5.00	20.00

NA, No activity observed: MIC or MBC of extracts were measured at 10.00 mg ml^−1^ and DMSO at 20%, respectively.

MIC = extract concentration at which the optical density (OD) measurement revealed minimal bacterial growth.

MBC = extract concentration at which a three-log reduction (99.9%) of the bacterial growth was induced.

In Figure 3, it is evident that the biofilm formation potential of *S. mutans* is significantly reduced at concentrations up to 1.25 mg/ml. Therefore, concentrations at or above this threshold can be categorized as C1, indicating strong inhibition of biofilm production. A concentration of 0.60 mg/ml still exhibited moderate biofilm production, while concentrations higher than 1.25 mg/ml were categorized as C3, indicating no detectable effect on biofilm formation.

The ethyl acetate extract of *M. longifolia* demonstrated overall effectiveness against all tested bacterial and fungal pathogens, as shown in Table 6. The minimum inhibitory concentration (MIC) values ranged from 0.08 mg/ml (*P. micra*) to 5.00 mg/ml (*E. coli*). Among the Streptococcus strains, the extract had the most significant effect on *S. oralis* and *S. mutans*, with an MIC value of 0.60 mg/ml. However, the MBC values indicated bactericidal effects within a range of 0.08 mg/ml (*P. micra*) to 5.00 mg/ml (*S. oralis*). No bactericidal or fungicidal effects were observed against *S. mutans*, *S. sobrinus*, *E. faecalis*, *C. albicans*, and *E. coli*.

**Table 6:** Antimicrobial activity in mg ml^−1^ of *Mentha longofolia* ethyl acetate extract.

Mentha longofolia				
Sample	Ethyl acetate extract DMSO (%)			
c/mg ml**^−^**^1^	MIC	MBC	MIC	MBC
*Streptococcus mutans* DSM 20523	0.60	NA	5.00	NA
*Streptococcus sobrinus* DSM 20381	1.25	NA	20.00	NA
*Streptococcus oralis* ATCC 35037	0.60	5.00	10.00	20
*Enterococcus faecalis* ATCC 29212	2.50	NA	20.00	NA
*Candida albicans* DSM 1386	2.50	NA	10.00	NA
*Escherichia coli* ATCC 25922	5.00	NA	20.00	NA
*Staphylococcus aureus* ATCC 25923	1.25	2.50	20.00	NA
*Porphyromonas gingivalis* W381	0.15	0.60	20.00	20.00
*Prevotella intermedia* MSP 34	0.30	0.60	5.00	5.00
*Fusobacterium nucleatum* ATCC 25586	0.60	1.25	10.00	10.00
*Parvimonas micra* ATCC 23195	0.08	0.08	5.00	20.00

NA, No activity observed: MIC or MBC of extracts were measured at 10.00 mg ml^−1^ and DMSO at 20%, respectively.

MIC = extract concentration at which the optical density (OD) measurement revealed minimal bacterial growth.

MBC = extract concentration at which a three-log reduction (99.9%) of the bacterial growth was induced.

**Figure 2:**
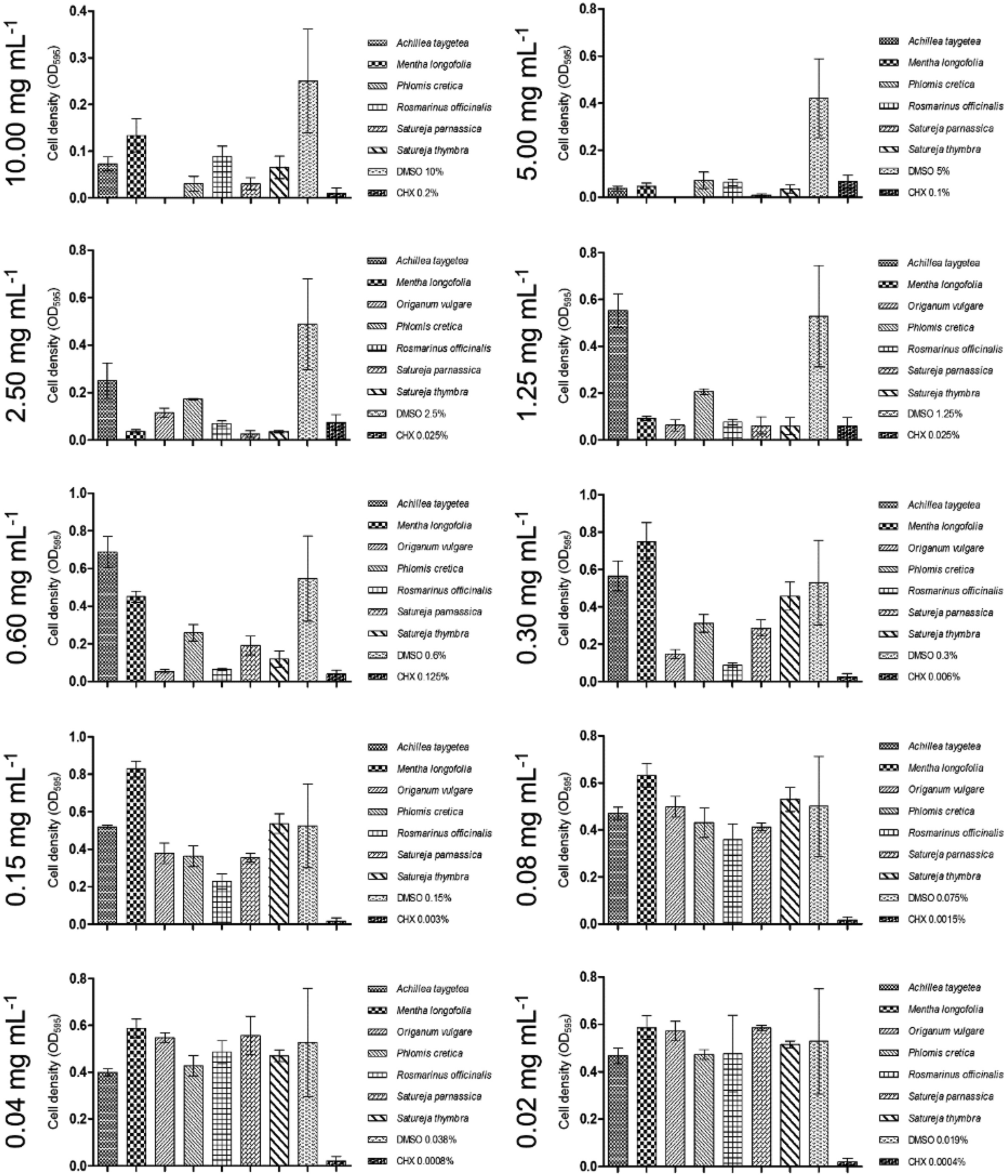
Graphs demonstrating ethyl acetate extracts with moderate impact on biofilm inhibition.

In the biofilm assay conducted with the tested strain of *S. mutans* (Figure 2), the ethyl acetate extract showed a moderate inhibitory effect on biofilm formation. Absorbance values correlated with the category of no biofilm production for concentrations as low as 1.25 mg/ml.

#### Origanum vulgare

The ethyl acetate extract demonstrated a broad spectrum of activity against all tested pathogens, particularly the oral bacteria (as shown in Table 7), with a MIC range of 0.04 mg/ml (*P. gingivalis*, *P. micra*) to 2.50 mg/ml (*E. coli*). For the facultative anaerobic bacteria, MIC values varied between 0.15 mg/ml (*S. mutans*, *S. sobrinus*, *S. oralis*) and 2.50 mg/ml (*E. coli*). The minimum bactericidal concentration (MBC) values ranged from 0.08 mg/ml (*P. micra*) to 2.50 mg/ml (*E. coli*, *C. albicans*). In comparison to *C. albicans* and *E. coli* (MBC = 2.50 mg/ml), all other pathogens were eliminated more effectively, with concentrations ranging from 0.08 mg/ml (*P. micra*) to 1.25 mg/ml (*E. faecalis*).

**Table 7:** Antimicrobial activity in mg ml^−1^ of *Origanum vulgare* ethyl acetate extract.

Origanum vulgare				
Sample	Ethyl acetate extract DMSO (%)			
c/mg ml**^−^**^1^	MIC	MBC	MIC	MBC
*Streptococcus mutans* DSM 20523	0.15	0.30	5.00	NA
*Streptococcus sobrinus* DSM 20381	0.15	0.15	20.00	20.00
*Streptococcus oralis* ATCC 35037	0.15	0.15	5.00	20.00
*Enterococcus faecalis* ATCC 29212	1.25	1.25	20.00	NA
*Candida albicans* DSM 1386	1.25	2.50	10.00	NA
*Escherichia coli* ATCC 25922	2.50	2.50	20.00	20.00
*Staphylococcus aureus* ATCC 25923	0.30	0.30	20.00	NA
*Porphyromonas gingivalis* W381	0.04	0.15	20.00	20.00
*Prevotella intermedia* MSP 34	0.30	0.60	5.00	5.00
*Fusobacterium nucleatum* ATCC 25586	0.15	0.15	10.00	10.00
*Parvimonas micra* ATCC 23195	0.04	0.08	2.50	10.00

NA, No activity observed: MIC or MBC of extracts were measured at 10.00 mg ml^−1^ and DMSO at 20%, respectively.

MIC = extract concentration at which the optical density (OD) measurement revealed minimal bacterial growth.

MBC = extract concentration at which a three-log reduction (99.9%) of the bacterial growth was induced.

In the biofilm plate assay, a moderate reduction in biofilm production was observed, as depicted in Figure 2. The lower cutoff value was established between a concentration of 0.30 mg/ml and 0.60 mg/ml, while high biofilm formation was observed at concentrations of 0.08 mg/ml and below.

#### Phlomis cretica

Table 8 presents the inhibitory effects of the ethyl acetate extract, particularly on the bacterial strains. When considering the impact of DMSO, the extract showed no significant effect on *E. faecalis*, *E. coli*, and *C. albicans*. The minimum inhibitory concentration (MIC) values ranged from 0.04 mg/ml (*P. micra*) to 2.50 mg/ml (*S. sobrinus*, *S. aureus*). The minimum bactericidal concentration (MBC) values indicated the persistence of *S. mutans*, *S. sobrinus*, *C. albicans*, *E. faecalis*, and *E. coli* in the presence of the ethyl acetate extract from *P. cretica*, while all other strains were killed at concentrations ranging from 0.08 mg/m (*P. micra*) to 2.50 mg/ml *(S. aureus*).

**Table 8:** Antimicrobial activity in mg ml^−1^ of *Phlomis cretica* ethyl acetate extract.

Phlomis cretica				
Sample	Ethyl acetate extract DMSO (%)			
c/mg ml**^−^**^1^	MIC	MBC	MIC	MBC
*Streptococcus mutans* DSM 20523	1.25	NA	5.00	NA
*Streptococcus sobrinus* DSM 20381	2.50	NA	20.00	NA
*Streptococcus oralis* ATCC 35037	0.30	0.30	10.00	20.00
*Enterococcus faecalis* ATCC 29212	NA	NA	20.00	NA
*Candida albicans* DSM 1386	10.00	NA	10.00	NA
*Escherichia coli* ATCC 25922	NA	NA	20.00	NA
*Staphylococcus aureus* ATCC 25923	2.50	2.50	20.00	NA
*Porphyromonas gingivalis* W381	0.15	0.60	20.00	20.00
*Prevotella intermedia* MSP 34	0.60	1.25	5.00	5.00
*Fusobacterium nucleatum* ATCC 25586	1.25	1.25	10.00	10.00
*Parvimonas micra* ATCC 23195	0.04	0.08	5.00	20.00

NA: No activity observed: MIC or MBC of extracts were measured at 10.00 mg ml^−1^ and DMSO at 20%, respectively.

MIC = extract concentration at which the optical density (OD) measurement revealed minimal bacterial growth.

MBC = extract concentration at which a three-log reduction (99.9%) of the bacterial growth was induced.

In the biofilm plate assay, no accumulated biofilm was observed in the presence of 5.00 mg/ml of the ethyl acetate extract. With over five dilution steps, including 0.15 mg/ml (as shown in Figure 2), a moderate biofilm formation was observed, while lower concentrations fell into category C3, indicating no detectable effect on biofilm formation.

#### Rosmarinus officinalis

The ethyl acetate extract of *R. officinalis* exhibited significant antibacterial effects against both oral facultative and obligate anaerobic bacteria, as shown in Table 9. The mean minimum inhibitory concentration (MIC) values for facultative anaerobic bacteria ranged from 0.02 mg/ml (*S. mutans*) to 0.60 mg/ml (*E. faecalis*), while for obligate anaerobes, the MIC values ranged from 0.01 mg/ml (*P. micra*) to 0.15 mg/ml (*F. nucleatum*). The extract also effectively reduced the growth of *S. aureus*, with MIC/MBC values of 0.30 mg/ml. However, it did not show any negative impact on *E. coli* and *C. albicans*. Notably, the minimum bactericidal concentration (MBC) values for oral bacteria ranged from 0.02 mg/m (*P. micra*) to 1.25 mg/ml (*E. faecalis*).

**Table 9:** Antimicrobial activity in mg ml^−1^ of *Rosmarinus officinalis* ethyl acetate extract.

Rosmarinus officinalis				
Sample	Ethyl acetate extract DMSO (%)			
c/mg ml**^−^**^1^	MIC	MBC	MIC	MBC
*Streptococcus mutans* DSM 20523	0.02	0.08	5.00	NA
*Streptococcus sobrinus* DSM 20381	0.08	0.15	20.00	20.00
*Streptococcus oralis* ATCC 35037	0.15	0.30	10.00	20.00
*Enterococcus faecalis* ATCC 29212	0.60	1.25	20.00	NA
*Candida albicans* DSM 1386	10.00	10.00	5.00	NA
*Escherichia coli* ATCC 25922	10.00	10.00	20.00	NA
*Staphylococcus aureus* ATCC 25923	0.30	0.30	20.00	NA
*Porphyromonas gingivalis* W381	0.04	0.08	20.00	20.00
*Prevotella intermedia* MSP 34	0.04	0.04	2.50	5.00
*Fusobacterium nucleatum* ATCC 25586	0.15	0.15	10.00	10.00
*Parvimonas micra* ATCC 23195	0.01	0.02	10.00	20.00

NA, No activity observed: MIC or MBC of extracts were measured at 10.00 mg ml^−1^ and DMSO at 20%, respectively.

MIC = extract concentration at which the optical density (OD) measurement revealed minimal bacterial growth.

MBC = extract concentration at which a three-log reduction (99.9%) of the bacterial growth was induced.

In the biofilm plate assay conducted with the tested strain of *S. mutans* (Figure 2), it was observed that the production of biofilm was suppressed at an extract concentration of 0.30 mg/ml. A concentration of 0.08 mg/ml was sufficient for moderate biofilm production, while concentrations of 0.04 mg/ml and lower fell into category C3, indicating no detectable effect on biofilm formation.

#### Salvia sclarea

Table 10 presents the inhibitory effect of the ethyl acetate extract on all obligate anaerobic bacteria. The MIC values ranged from 0.04 mg/ml (*P. gingivalis*, *P. micra*) to 0.15 mg/ml (*F. nucleatum*). Some other bacterial strains were inhibited at concentrations ranging from 0.15 mg/ml (*S. oralis*) to 5.00 mg/ml (*S*. *sobrinus*). The MBC values demonstrated a fatal reduction in bacterial growth, ranging from 0.08 mg/ml (*P. gingivalis*, *P. intermedia*, *P. micra*) to 10.00 mg/ml (*S. sobrinus*), with no measurable bactericidal reduction observed for *E. faecalis*. In terms of the impact of DMSO, the extract did not show noteworthy effects on *S. mutans*, *E. coli*, and *C. albicans*.

**Table 10:** Antimicrobial activity in mg ml^−1^ of *Sal*via *sclarea* ethyl acetate extract.

Salvia sclarea				
Sample	Ethyl acetate extract DMSO (%)			
c/mg ml**^−^**^1^	MIC	MBC	MIC	MBC
*Streptococcus mutans* DSM 20523	5.00	NA	10.00	NA
*Streptococcus sobrinus* DSM 20381	5.00	10.00	20.00	NA
*Streptococcus oralis* ATCC 35037	0.15	0.15	20.00	20.00
*Enterococcus faecalis* ATCC 29212	2.50	NA	20.00	NA
*Candida albicans* DSM 1386	10.00	10.00	10.00	NA
*Escherichia coli* ATCC 25922	NA	NA	20.00	NA
*Staphylococcus aureus* ATCC 25923	0.60	1.25	20.00	NA
*Porphyromonas gingivalis* W381	0.04	0.08	20.00	20.00
*Prevotella intermedia* MSP 34	0.08	0.08	5.00	5.00
*Fusobacterium nucleatum* ATCC 25586	0.15	0.15	10.00	10.00
*Parvimonas micra* ATCC 23195	0.04	0.08	5.00	20.00

NA, No activity observed: MIC or MBC of extracts were measured at 10.00 mg ml^−1^ and DMSO at 20%, respectively.

MIC = extract concentration at which the optical density (OD) measurement revealed minimal bacterial growth.

MBC = extract concentration at which a three-log reduction (99.9%) of the bacterial growth was induced.

**Figure 3:**
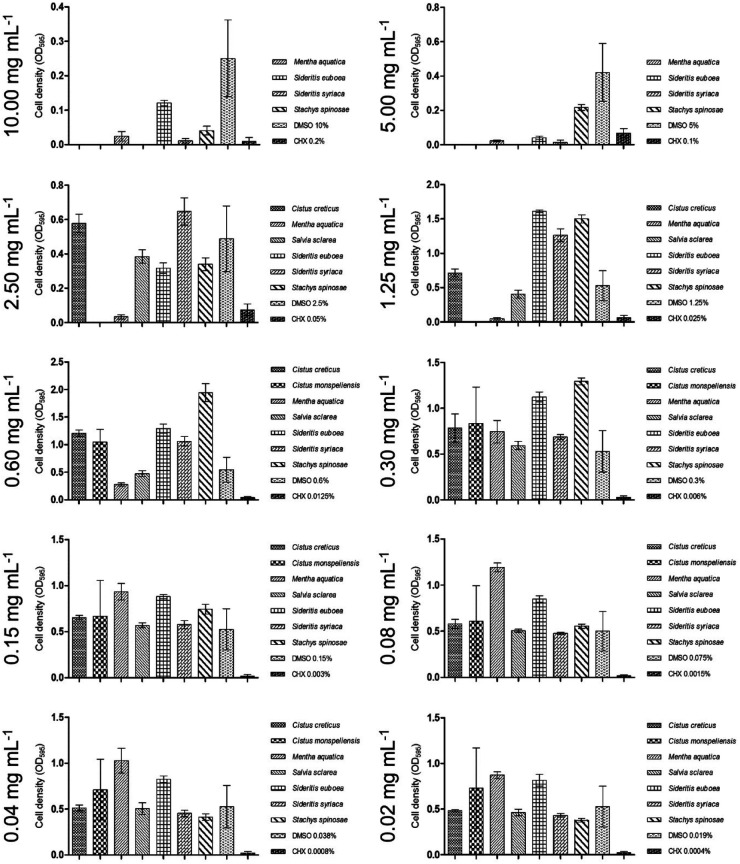
Graphs demonstrating ethyl acetate extracts with low impact on biofilm inhibition.

Figure 3 indicates that the ethyl acetate extract of Salvia sclarea had no detectable effect on biofilm accumulation.

#### Satureja parnassica and Satureja thymbra

Table 11 provides the inhibitory effects of the ethyl acetate extract on all bacterial strains (Table 12). The MIC values ranged from 0.08 mg/ml (*P. gingivalis*) to 5.00 mg/ml (*E. coli*, *S. aureus*). However, *C. albicans* was not affected by the extract from *S. parnassica*. Moreover, the extract exhibited bactericidal effects, with 99.9% of obligate anaerobic bacteria, *S. aureus*, *S. oralis*, and *S. sobrinus* being killed at concentrations ranging from 0.60 mg/ml (*P. micra*) to 5.00 mg/ml (*S. aureus*).

**Table 11:** Antimicrobial activity in mg ml^−1^ of *Satureja parnassica* ethyl acetate extract.

Satureja parnassica				
Sample	Ethyl acetate extract DMSO (%)			
c/mg ml**^−^**^1^	MIC	MBC	MIC	MBC
*Streptococcus mutans* DSM 20523	0.60	NA	10.00	NA
*Streptococcus sobrinus* DSM 20381	2.50	2.50	20.00	NA
*Streptococcus oralis* ATCC 35037	0.60	1.25	10.00	20.00
*Enterococcus faecalis* ATCC 29212	2.50	NA	20.00	NA
*Candida albicans* DSM 1386	5.00	NA	10.00	20.00
*Escherichia coli* ATCC 25922	5.00	NA	20.00	NA
*Staphylococcus aureus* ATCC 25923	5.00	5.00	20.00	NA
*Porphyromonas gingivalis* W381	0.08	1.25	20.00	20.00
*Prevotella intermedia* MSP 34	0.60	1,25	5.00	5.00
*Fusobacterium nucleatum* ATCC 25586	1.25	2.50	10.00	10.00
*Parvimonas micra* ATCC 23195	0.30	0.60	5.00	20.00

NA, No activity observed: MIC or MBC of extracts were measured at 10.00 mg ml^−1^ and DMSO at 20%, respectively.

MIC = extract concentration at which the optical density (OD) measurement revealed minimal bacterial growth.

MBC = extract concentration at which a three-log reduction (99.9%) of the bacterial growth was induced.

**Table 12:** Antimicrobial activity in mg ml^−1^ of *Satureja thymbra* ethyl acetate extract.

Satureja thymbra				
Sample	Ethyl acetate extract DMSO (%)			
c/mg ml**^−^**^1^	MIC	MBC	MIC	MBC
*Streptococcus mutans* DSM 20523	1.25	5.00	5.00	NA
*Streptococcus sobrinus* DSM 20381	0.60	1.25	20.00	NA
*Streptococcus oralis* ATCC 35037	0.30	0.30	10.00	20.00
*Enterococcus faecalis* ATCC 29212	1.25	5.00	20.00	NA
*Candida albicans* DSM 1386	5.00	10.00	10.00	NA
*Escherichia coli* ATCC 25922	10.00	10.00	20.00	20.00
*Staphylococcus aureus* ATCC 25923	0.60	0.60	20.00	NA
*Porphyromonas gingivalis* W381	0.08	0.15	20.00	20.00
*Prevotella intermedia* MSP 34	0.60	0.60	5.00	5.00
*Fusobacterium nucleatum* ATCC 25586	0.30	0.30	5.00	10.00
*Parvimonas micra* ATCC 23195	0.04	0.08	2.50	20.00

NA, No activity observed: MIC or MBC of extracts were measured at 10.00 mg ml^−1^ and DMSO at 20%, respectively.

MIC = extract concentration at which the optical density (OD) measurement revealed minimal bacterial growth.

MBC = extract concentration at which a three-log reduction (99.9%) of the bacterial growth was induced.

In the biofilm plate assay, the extract was able to inhibit biofilm formation at a concentration of 1.25 mg/ml. Furthermore, concentrations ranging from 0.60 mg/ml to 0.08 mg/ml resulted in moderate biofilm production, as shown in Figure 2.

The ethyl acetate extract of *S. thymbra* demonstrated effectiveness against nearly all tested microorganisms, with a mean minimum inhibitory concentration (MIC) range of 0.04 mg/ml (*P. micra*) to 1.25 mg/ml (*S. mutans*, *E. faecalis*). For the facultative anaerobic bacteria, MIC values varied from 0.30 mg/ml (*S. oralis*) to 1.25 mg/ml (*E. faecalis*, *S. mutans*). The minimum bactericidal concentration (MBC) values of the *S. thymbra* ethyl acetate extract ranged from 0.08 mg/ml (*P. micra*) to 5.00 mg/ml (*S. mutans*, *E. faecalis*). However, no antibacterial effects were observed on *E. coli*, and no antifungal effects were observed on *C. albicans* in relation to DMSO effects.

In the biofilm plate assay conducted with the tested strain of *S. mutans* (Figure 2), the ethyl acetate extract exhibited a moderate inhibitory effect on biofilm formation. Below a concentration of 0.60 mg/ml, the absorbance values correlated with the category of no biofilm production.

### Sideritis Euboea and Sideritis syriaca

Table 13 provides the MIC and MBC values for the ethyl acetate extract against all tested bacterial and fungal strains. The MIC values ranged from 0.15 mg/ml (*P. gingivalis*, *P. intermedia*, *P. micra*) to 2.50 mg/ml (*S. mutans*, *S. sobrinus*). For 99.9% bacterial strain reduction, concentrations ranged from 0.15 mg/ml (*P. gingivalis*) to 2.50 mg/ml (*S. aureus*). However, *E. coli*, *E. faecalis*, and *C. albicans* did not appear to be significantly affected by the extract. Additionally, *S. mutans* and *S. sobrinus* were not eradicated by the tested concentrations of the *S. euboea* extract.

**Table 13:** Antimicrobial activity in mg ml^−1^ of *Sideritis euboea* ethyl acetate extract.

Sideritis euboea				
Sample	Ethyl acetate extract DMSO (%)			
c/mg ml**^−^**^1^	MIC	MBC	MIC	MBC
*Streptococcus mutans* DSM 20523	2.50	NA	10.00	NA
*Streptococcus sobrinus* DSM 20381	2.50	NA	20.00	NA
*Streptococcus oralis* ATCC 35037	0.60	0.60	10.00	20.00
*Enterococcus faecalis* ATCC 29212	NA	NA	20.00	NA
*Candida albicans* DSM 1386	10.00	10.00	10.00	NA
*Escherichia coli* ATCC 25922	NA	NA	20.00	NA
*Staphylococcus aureus* ATCC 25923	1.25	2.50	20.00	NA
*Porphyromonas gingivalis* W381	0.15	0.15	20.00	20.00
*Prevotella intermedia* MSP 34	0.15	0.30	5.00	5.00
*Fusobacterium nucleatum* ATCC 25586	0.60	0.60	10.00	10.00
*Parvimonas micra* ATCC 23195	0.15	0.30	5.00	20.00

NA, No activity observed: MIC or MBC of extracts were measured at 10.00 mg ml^−1^ and DMSO at 20%, respectively.

MIC = extract concentration at which the optical density (OD) measurement revealed minimal bacterial growth.

MBC = extract concentration at which a three-log reduction (99.9%) of the bacterial growth was induced.

In the biofilm plate assay, the minimal concentration required to counteract biofilm production was determined to be 5.00 mg/ml, as shown in Figure 3. Concentrations of at least 2.50 mg/ml were categorized as C2, while lower concentrations did not influence biofilm formation (C3).

In comparison to the *S. euboea* extract, the extract of *S. syriaca* exhibited stronger inhibition against *S. sobrinus*, *E. faecalis*, *P. gingivalis*, *P. micra*, *S. aureus*, and *E. coli* (Table 14). Mean MIC values ranged between 0.08 mg/ml (*P. gingivalis*, *P. micra*) and 2.50 mg/ml (*E. coli*, *E. faecalis*). *S. mutans* and *C. albicans* were not suppressed by the extract. Obligate anaerobes and *S. oralis* were eliminated more easily compared to *S. aureus*, *S. mutans*, and *S. sobrinus*, with a range of 0.08 mg/ml (*P. micra*) to 10.00 mg/ml (*S. mutans*, *S. sobrinus*).

**Table 14:** Antimicrobial activity in mg ml^−1^ of *Sideritis syriaca* ethyl acetate extract.

Sideritis syriaca				
Sample	Ethyl acetate extract DMSO (%)			
c/mg ml**^−^**^1^	MIC	MBC	MIC	MBC
*Streptococcus mutans* DSM 20523	2.50	10.00	5.00	NA
*Streptococcus sobrinus* DSM 20381	1.25	10.00	20.00	NA
*Streptococcus oralis* ATCC 35037	0.60	0.60	10.00	20.00
*Enterococcus faecalis* ATCC 29212	2.50	NA	20.00	NA
*Candida albicans* DSM 1386	5.00	NA	10.00	NA
*Escherichia coli* ATCC 25922	2.50	NA	20.00	NA
*Staphylococcus aureus* ATCC 25923	0.60	2.50	20.00	NA
*Porphyromonas gingivalis* W381	0.08	0.15	20.00	20.00
*Prevotella intermedia* MSP 34	0.15	0.30	5.00	5.00
*Fusobacterium nucleatum* ATCC 25586	0.60	0.60	10.00	10.00
*Parvimonas micra* ATCC 23195	0.08	0.08	5.00	20.00

NA, No activity observed: MIC or MBC of extracts were measured at 10.00 mg ml^−1^ and DMSO at 20%, respectively.

MIC = extract concentration at which the optical density (OD) measurement revealed minimal bacterial growth.

MBC = extract concentration at which a three-log reduction (99.9%) of the bacterial growth was induced.

As shown in Figure 3, *S. mutans* biofilm production was completely inhibited at 5.00 mg/ml of the *S. syriaca* extract (C1).

#### Stachys spinosa

The extract exhibited inhibitory effects against all oral bacteria and *S. aureus*, with MIC values ranging from 0.15 mg/ml (*P. micra*) to 2.50 mg/ml (*S. oralis*, *E. faecalis*, *S. aureus*). However, notable bactericidal effects were observed only for obligate anaerobes (MBC of *P. micra*: 0.30 mg/ml), *S. oralis* (5.00 mg/ml), and *S. aureus* (10.00 mg/ml, as shown in Table 15.

**Table 15:** Antimicrobial activity in mg ml^−1^ of *Stachys spinosae* ethyl acetate extract.

Stachys spinosae				
Sample	Ethyl acetate extract DMSO (%)			
c/mg ml**^−^**^1^	MIC	MBC	MIC	MBC
*Streptococcus mutans* DSM 20523	0.60	NA	10.00	NA
*Streptococcus sobrinus* DSM 20381	0.60	NA	20.00	NA
*Streptococcus oralis* ATCC 35037	2.50	5.00	10.00	20.00
*Enterococcus faecalis* ATCC 29212	2.50	NA	20.00	NA
*Candida albicans* DSM 1386	NA	NA	10.00	NA
*Escherichia coli* ATCC 25922	10.00	NA	20.00	NA
*Staphylococcus aureus* ATCC 25923	2.50	10.00	20.00	NA
*Porphyromonas gingivalis* W381	0.30	0.60	20.00	20.00
*Prevotella intermedia* MSP 34	1.25	1.25	5.00	5.00
*Fusobacterium nucleatum* ATCC 25586	1.25	1.25	10.00	10.00
*Parvimonas micra* ATCC 23195	0.15	0.30	5.00	20.00

NA, No activity observed: MIC or MBC of extracts were measured at 10.00 mg ml^−1^ and DMSO at 20%, respectively.

MIC = extract concentration at which the optical density (OD) measurement revealed minimal bacterial growth.

MBC = extract concentration at which a threelog reduction (99.9%) of the bacterial growth was induced.

No biofilm production was detected at an extract concentration of 10.00 mg/ml. However, concentrations as low as 2.50 mg/ml were sufficient to inhibit biofilm formation, categorized as C2 (Figure 3).

#### Thymus longicaulis

Despite of *E. coli* and *C. albicans*, which weren't restricted, all pathogens were inhibited between 0.04 mg/ml (*P. gingivalis*) and 2.50 mg/ml (*S. sobrinus*, *S. aureus*). MBC values didn't approve the great effect as only *S. oralis* (1.25 mg/ml), obligate anaerobe growing bacteria (0.30 mg/ml - 2.50 mg/ml) and *S. aureus* (10.00 mg/ml) were killed at 99.9%, Table 16).

**Table 16:** Antimicrobial activity in mg ml^−1^ of *Thymus longicaulis* ethyl acetate extract.

Thymus longicaulis				
Sample	Ethyl acetate extract DMSO (%)			
c/mg ml**^−^**^1^	MIC	MBC	MIC	MBC
*Streptococcus mutans* DSM 20523	0.30	NA	5.00	NA
*Streptococcus sobrinus* DSM 20381	2.50	NA	20.00	NA
*Streptococcus oralis* ATCC 35037	0.30	1.25	10.00	20.00
*Enterococcus faecalis* ATCC 29212	1.25	NA	20.00	NA
*Candida albicans* DSM 1386	10.00	NA	10.00	20.00
*Escherichia coli* ATCC 25922	NA	NA	20.00	20.00
*Staphylococcus aureus* ATCC 25923	2.50	10.00	20.00	NA
*Porphyromonas gingivalis* W381	0.04	1.25	20.00	20.00
*Prevotella intermedia* MSP 34	1.25	2.50	5.00	5.00
*Fusobacterium nucleatum* ATCC 25586	1.25	2.50	10.00	10.00
*Parvimonas micra* ATCC 23195	0.15	0.30	2.50	10.00

NA, No activity observed: MIC or MBC of extracts were measured at 10.00 mg ml^−1^ and DMSO at 20%, respectively.

MIC = extract concentration at which the optical density (OD) measurement revealed minimal bacterial growth.

MBC = extract concentration at which a three-log reduction (99.9%) of the bacterial growth was induced.

Similarly, to the mentioned inhibitory effect on *S. mutans*, the biofilm plate assay showed no biofilm production up to an extract concentration of 0.30 mg/ml. Moreover, 0.04 mg/ml was sufficient to regulate biofilm down in a moderate spectrum and, presented in Figure 1, finally 0.02 mg/ml were classified C3.

## Discussion

The objective of this study was to evaluate the antimicrobial efficacy and ability to inhibit biofilm formation of 16 Mediterranean herb extracts against eight common oral bacterial pathogens and the fungus *C. albicans*. Previous research in the literature primarily focused on investigating the antimicrobial activity of essential oils derived from these extracts against non-oral bacteria and fungi, with only a couple of studies reporting on their antibiofilm properties against a single oral bacterial strain (30). We chose ethyl acetate extracts based on their ability to selectively isolate bioactive compounds with potential antimicrobial and antibiofilm properties while also being less toxic compared to solvents like methanol or ethanol. To the best of our knowledge, this is the first study to examine the antimicrobial and antibiofilm activities of ethyl acetate extracts from the aforementioned herb species against a variety of oral pathogens.

The ethyl acetate extract of *A. taygetea* demonstrated significant inhibition of obligate anaerobic bacteria. In comparison to the essential oil (EO) of *A. taygetea*, the ethyl acetate extract exhibited stronger inhibitory effects on Gram-positive facultative anaerobic bacteria than on Gram-negative bacteria (31).

To date, various studies (32–34) have confirmed the extended antimicrobial activity of *Cistus* spp. against diverse “non-oral” bacteria and fungi.

Gram-negative bacteria, being equipped with an outer cell membrane, pose a dense permeability barrier that restricts the entry of lipophilic molecules, rendering them more resistant to *Cistus* spp. extracts (35), compared to Gram-positive microorganisms (36). Gram-positive bacteria employ defense mechanisms such as extracellular protease production and chemical modifications of cell membranes or cell walls, which enhance their resistance to antimicrobial agents (37, 38). The use of Cistus tea for rinsing the oral cavity has been shown to reduce adherent bacteria on enamel surfaces *in situ* (39). Fungi, even after the application of high-concentrated extracts, could not be effectively killed. In contrast, *C. albicans*, as a representative fungus, could not be effectively eliminated even with high-concentration *Cistus* spp. extracts (32–34).

*S. euboea* has shown only moderate antimicrobial activity compared to other *Sideritis* spp. On the other hand, *S. syriaca* has been studied for its antibacterial properties, both as a decoction and as an essential oil. Despite their different compositions due to polarity, both forms have exhibited activity. The polar decoction contains components such as hypoelatin, isoscutellarein diglucosides, and chlorogenic acid, which contribute to its inhibitory effect on both Gram-positive and Gram-negative bacteria.

Several studies have investigated the antibacterial activity of *L. stoechas* essential oil against both Gram-positive and Gram-negative bacteria (40–42). However, a study specifically focused on oral bacteria found that the essential oil had limited effectiveness, with an MIC of 4 µl/ml (43).

When comparing different forms of *O. vulgare* extracts, such as decoction, infusion, and methanol-water extract, with varying amounts of compounds including luteolin O-glucuronide, luteolin 7-O-glucoside, and rosmarinic acid, it was observed that the ethyl acetate extract exhibited enhanced antibacterial activity against Gram-negative bacteria compared to Gram-positive microorganisms (44). Leaves of *O. vulgare* species collected in Mexico contained higher levels of *α*-pinene and terpinen-4-ol than thymol and carvacrol (45).

The tested oral bacteria showed sensitivity to the rosemary extract, which is consistent with the findings of a study by Takarada et al. (46)using rosemary essential oil (EO). Rosemary leaves were found to contain higher levels of inhibitory compounds compared to stems. The main components carnosic acid and carnosol exhibited MIC values of 0.09 mg/ml and 0.08 mg/ml against *S. mutans* and *S. sobrinus*, respectively. They also demonstrated eradication of *E. faecalis* at concentrations of 0.07 mg/ml and 0.10 mg/ml, respectively (47). Significantly, a polyherbal mouthwash containing *R. officinalis* extract, among other hydroalcoholic extracts, demonstrated high antibacterial efficacy comparable to 0.2% (w/v) chlorhexidine (CHX) in the treatment of gingivitis in a randomized double-blind placebo-controlled trial (48). Another clinical study on periodontitis showed that a mouthrinse containing *Rosmarinus* spp. essential oils, including rosemary, supported the eradication of subgingival biofilm primarily composed of obligate anaerobes (49). The potential mechanism behind this could be the inhibition of quorum sensing (QS) signals by rosemary compounds.

In the present study, the *S. sclarea* ethyl acetate extract exhibited high antibacterial activity against obligate anaerobic oral pathogens, surpassing the activity of the EO (50). Notably, *S. sclarea* has been shown to inhibit the growth of methicillin-resistant *Staphylococcus epidermidis* when combined with oxacillin, potentially through the action of diterpenes that inhibit the expression of penicillin-binding proteins (PBPs) (51).

The ethyl acetate extract of *M. aquatica* exhibited weak inhibitory effects on facultative anaerobic bacteria, which aligns with the findings of an essential oil (EO) study that showed limited activity against *E. coli* and *S. aureus* strains, as well as minimal effect on *C. albicans (*52). It seems that the antimicrobial activity of *M. longifolia* is not solely dependent on the higher amount of monoterpene hydrocarbons, but rather on a balanced combination of monoterpene hydrocarbons and oxygenated monoterpenes (53).

In a comparative study of *M. longifolia* ethyl acetate and aqueous extracts, the ethyl acetate extract demonstrated slightly stronger bactericidal effects against *S. aureus* (54), which is consistent with our findings. Interestingly, coccoid-shaped bacteria, such as *S. aureus*, tend to show less cell damage at MIC values compared to rod-shaped bacteria like *E. coli*. Although the ethyl acetate extract of *M. longifolia* inhibited the growth of *S. mutans* in the current study, it did not completely eradicate the bacteria, unlike a hydroalcoholic extract that achieved a MBC value of 0.1 mg/ml, as reported by Kermanshah et al. (55). Overall, the extract of *M. longifolia* demonstrated stronger inhibitory effects compared to the *M. aquatica* extract, consistent with the findings of Mimica-Dukić et al. (52).

The antimicrobial activity of the ethyl acetate extract of *S. spinosa* has not been previously investigated. However, the observed inhibitory effects of the extract on various bacterial species can potentially be attributed to terpenes such as thymol and carvacrol.

The ethyl acetate extract of *P. cretica* exhibited inhibitory effects on the growth of *S. oralis* and obligate anaerobic bacteria. These results are consistent with a previous study that used an EO of *P. cretica* and reported relatively high MIC values for *S. aureus* and *E. coli*. The observed trend in our study may be attributed, among other factors, to the presence of *α*-pinene in the extract, which has been shown to have an impact on the growth of these bacterial strains, rather than caryophyllene (56).

Investigating the impact of plant collection time on EO activity, it was observed that both *S. parnassica* and *S. thymbra* collected in full flower had the lowest MIC values against the foodborne pathogens *Salmonella enterica* and *Listeria monocytogenes* (57). It is worth noting that the relative proportions of carvacrol and thymol, rather than their absolute quantities, seem to play a role in determining the activity, with an optimum ratio near 3:2 (carvacrol:thymol). The ethyl acetate extract of *S. thymbra* exhibited stronger inhibition against Gram-positive bacteria such as *S. aureus* and *E. faecalis* compared to the Gram-negative bacterium *E. coli*. This finding is not fully consistent with the EOs tested by Giweli *et al*. (58), which showed slightly lower MIC values against *S. aureus* compared to *E. coli*.

The ethyl acetate extract of *T. longicaulis* exhibited significant inhibitory activity against Gram-positive bacteria, including *S. aureus* and *S. mutans*. However, this aromatic herb also showed activity against Gram-negative bacteria, as demonstrated with an essential oil in a study by De Martino *et al*. (59), which examined herbs from two different regions. Interestingly, the EO with higher quantities of thymol and carvacrol, and nearly a 2:3 ratio, exhibited weaker inhibition against all strains. In general, encapsulating extracts may be a promising technique to enhance their effectiveness, as indicated by the comparison of the more active methanolic extract to the dichloromethane extract (60).

The available studies on the antibiofilm activity of the tested plant species are limited but provide valuable insights (30, 61, 62). For example, the components salvipisone and aethiopinone from *S. sclarea* effectively reduced biofilm quantities produced by *S. aureus* and *S. epidermidis* (61). Methicillin-resistant *S. aureus* biofilm was also reduced by a rosemary ethanolic extract (62). Even in ten-fold lower concentrations than CHX, rosemary EO as a toothpaste component had higher antibiofilm formation activity against *S. mutans* (30).

Variability in MIC values is not uncommon in microdilution testing according to the Clinical and Laboratory Standards Institute (CLSI) in microbiological practice. Changes by a factor of two are acceptable for EUCAST [European Committee on Antimicrobial Susceptibility Testing. MIC distributions and epidemiological cut-off value (ECOFF) setting, EUCAST SOP 10.2, 2021]. This also depends on the microbial culture used and the day of use. The MIC values are therefore given as a range for many antimicrobial substances. In our results, the MIC value of DMSO only changed by a factor of two for a few microorganisms. Furthermore, this inhibition value was always taken into account to assess the value of the MIC results obtained for the various plant extracts.

Our study aimed to verify whether plant extracts inhibit biofilm formation, using the crystal violet staining method, as done in previous studies. The MTT test could assess the activity of already formed biofilm, providing an interesting future research approach to evaluate the antimicrobial effects of these extracts.We tested mono-species biofilms to establish a controlled baseline for evaluating the inhibitory effects of plant extracts on *S. mutans*, allowing us to attribute any observed impacts directly to the extracts without the confounding influence of interspecies interactions that can complicate multi-species biofilm assessments.

In summary, the outcomes of this investigation underscore the potent inhibitory capabilities of the Mediterranean herbs under scrutiny against the assessed obligate anaerobic microorganisms found in the oral environment. These findings suggest a promising avenue for developing these herbs into natural agents with antimicrobial and antibiofilm properties, particularly targeted against oral pathogens. Potential antibiofilm mechanisms of the tested extracts mechanisms include interference with quorum sensing, disruption of extracellular polymeric substance (EPS) production, and inhibition of bacterial adhesion (63–65). Future studies could explore whether these effects occur at subinhibitory concentrations and assess their impact on acid tolerance and production in cariogenic bacteria. The specific composition of compounds within the herbs exerts a pivotal influence on their antimicrobial efficacy, thereby necessitating careful consideration of factors such as collection timing, geographical origin, and extraction methodologies. The growing tolerance to CHX underscores the need for exploring alternative antimicrobial and antibiofilm agents, such as the plant extracts investigated in our study. Future research could further evaluate these extracts' long-term effects and resistance profiles compared to conventional antiseptics.

Remarkably, the ethyl acetate extracts derived from *Rosmarinus officinalis* and *Origanum vulgare* exhibited noteworthy antimicrobial effects against the entire spectrum of oral pathogens examined. Furthermore, the *Lavandula stoechas* extract demonstrated marked potential in countering biofilm formation by *S. mutans*. The strategic combination of these plant extracts could conceivably serve as a foundational element in alternative antibacterial formulations, thereby contributing to the mitigation of biofilm-associated oral afflictions like caries and periodontitis.

## Data Availability

The original contributions presented in the study are included in the article/Supplementary Material, further inquiries can be directed to the corresponding author.
