# Supplementary figures and images for: Unleashing nature's defense: potent antimicrobial power of plant extracts against oral pathogens and Streptococcus mutans biofilms
Source: Front Oral Health. 2024 Dec 12;5:1469174. doi: 10.3389/froh.2024.1469174 (PMC11669686; doi:10.3389/froh.2024.1469174)

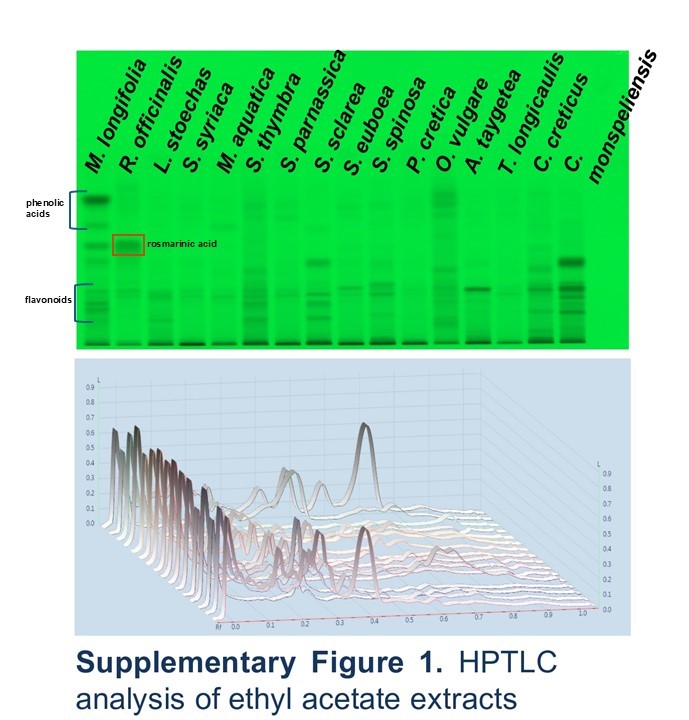

Supplement: Supplementary file 1 [file Image1.jpeg]
